# Persistent atherogenic lipoprotein changes during lorlatinib therapy

**DOI:** 10.1210/jcemcr/luag324

**Published:** 2026-10-22

**Authors:** Kimitaka Shibue, Kei Kunimasa, Akihiro Hamasaki

**Affiliations:** Department of Diabetes and Endocrinology, Medical Research Institute KITANO HOSPITAL, PIIF Tazuke-Kofukai, Osaka 530-8480, Japan; Department of Thoracic Oncology, Osaka International Cancer Institute, Osaka 541-8567, Japan; Department of Diabetes and Endocrinology, Medical Research Institute KITANO HOSPITAL, PIIF Tazuke-Kofukai, Osaka 530-8480, Japan

**Keywords:** lorlatinib, drug-induced dyslipidemia, hypertriglyceridemia, apolipoproteins, lipoprotein metabolism

## Abstract

Lorlatinib, a third-generation anaplastic lymphoma kinase (ALK)/ROS1 tyrosine kinase inhibitor, frequently induces hypercholesterolemia and hypertriglyceridemia, but longitudinal qualitative lipoprotein changes remain incompletely characterized. We describe a 52-year-old woman with ALK-rearranged non–small cell lung cancer who developed persistent dyslipidemia despite lipid-lowering therapy. Before lorlatinib, total cholesterol was 164 mg/dL (SI [International System of Units]: 4.24 mmol/L) (reference range, 148-248 mg/dL [SI: 3.83-6.41 mmol/L]), low-density lipoprotein (LDL) cholesterol was 52 mg/dL (SI: 1.34 mmol/L) (reference range, 65-139 mg/dL [SI: 1.68-3.59 mmol/L]), and triglycerides were 54 mg/dL (SI: 0.61 mmol/L) (reference range, 30-117 mg/dL [SI: 0.34-1.32 mmol/L]). Within 3 weeks after lorlatinib initiation, these increased to 340, 245, and 166 mg/dL, respectively. Longitudinal profiling showed persistent apolipoprotein B (ApoB) elevation, increased oxidized LDL, and a prominent pre-β-lipoprotein fraction. Subclinical hypothyroidism was later detected, and pitavastatin was switched to rosuvastatin. This case highlights the potential value of ApoB and qualitative lipid assessment during prolonged lorlatinib therapy.

## Introduction

Lorlatinib is a brain-penetrant, third-generation anaplastic lymphoma kinase (ALK)/ROS1 tyrosine kinase inhibitor used for ALK-rearranged non–small cell lung cancer, with demonstrated efficacy in the first-line setting [1-3]. Dyslipidemia is among its most frequent adverse effects and is more characteristic of lorlatinib than of earlier ALK inhibitors, often requiring lipid-lowering therapy [3-5]. Reported lipid abnormalities include hypercholesterolemia and hypertriglyceridemia, typically developing within the first few weeks of treatment [4, 5].

Current clinical monitoring recommendations primarily rely on total cholesterol, low-density lipoprotein cholesterol (LDL-C), high-density lipoprotein cholesterol (HDL-C), and triglycerides [4, 5]. A recent case study emphasized that lorlatinib can induce complex dyslipidemia [6], and lipidology literature supports the use of apolipoprotein B (ApoB) and non-HDL-C as complementary markers of atherogenic lipoprotein burden [7]. However, longitudinal data describing ApoB, oxidized LDL, and lipoprotein electrophoretic patterns during lorlatinib therapy remain limited [4-6].

## Case presentation

A 52-year-old woman presented with cough and was found to have a right lower lobe lung mass on chest computed tomography. Bronchoscopic biopsy demonstrated thyroid transcription factor-1–positive adenocarcinoma, and staging investigations revealed mediastinal lymph node metastases and pleural dissemination, consistent with stage IVA ALK-rearranged non–small cell lung cancer (Fig. 1).

**Figure 1:**
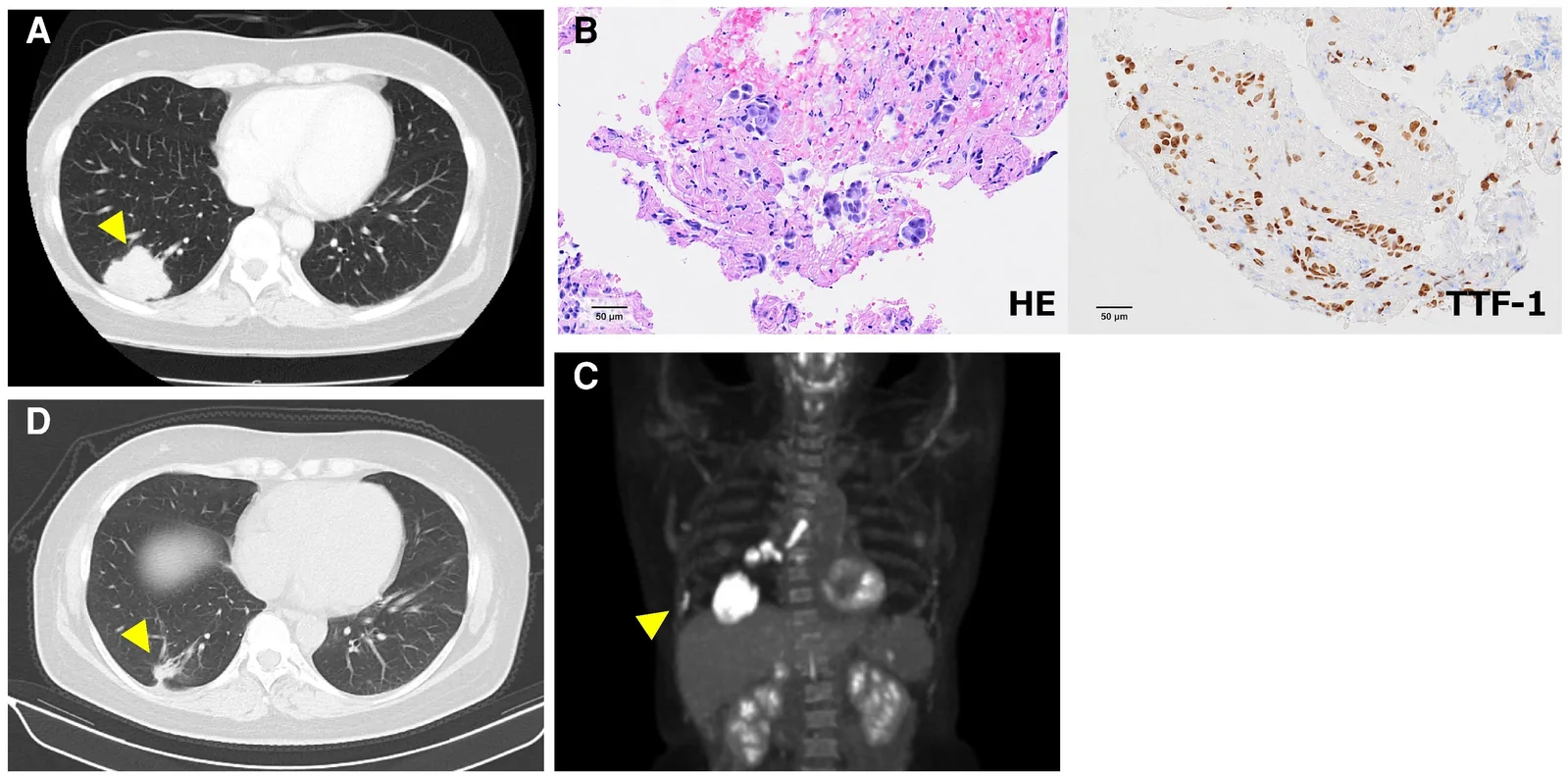
Radiologic and pathologic findings of the primary lung lesion. Chest images of the thoracic mass lesion and pathologic findings from the biopsy. A plain chest computed tomography image showed a mass lesion in the right lower lobe (A, arrow). Hematoxylin and eosin staining and thyroid transcription factor-1 immunohistochemical staining of the tumor biopsy specimen are shown (B; original magnification, ×200 for both images). Fluorodeoxyglucose uptake was observed in the right lower lobe mass and mediastinal lymph nodes, with additional uptake in the pleura (C, arrowhead).

For longitudinal analyses, day 0 was defined as the first lipid assessment at our outpatient clinic. Lorlatinib 100 mg once daily was initiated as first-line therapy on day 410. The patient had mild dyslipidemia treated with pitavastatin 4 mg daily before lorlatinib initiation. No other lipid-lowering therapy was used before day 434. At the closest available prelorlatinib lipid assessment on day 392, total cholesterol was 164 mg/dL (SI: 4.24 mmol/L) (reference range, 148-248 mg/dL [SI: 3.83-6.41 mmol/L]), triglycerides were 54 mg/dL (SI: 0.61 mmol/L) (reference range, 30-117 mg/dL [SI: 0.34-1.32 mmol/L]), HDL-C was 102 mg/dL (SI: 2.64 mmol/L) (reference range, 48-103 mg/dL [SI: 1.24-2.66 mmol/L]), and directly measured LDL-C was 52 mg/dL (SI: 1.34 mmol/L) (reference range, 65-139 mg/dL [SI: 1.68-3.59 mmol/L]) (Table 1). These are institutional laboratory reference ranges for reporting and differ from guideline-based lipid treatment thresholds used for cardiovascular risk management.

**Table 1:** Representative conventional lipid parameters during lorlatinib therapy

Day	Treatment status/change	TC (SI)	TG (SI)	HDL-C (SI)	LDL-C (SI)	Non-HDL-C (SI)
Day 392	P4; closest prelorlatinib	164 mg/dL (SI: 4.24 mmol/L)	54 mg/dL (SI: 0.61 mmol/L)	102 mg/dL (SI: 2.64 mmol/L)	52 mg/dL (SI: 1.34 mmol/L)	62 mg/dL (SI: 1.60 mmol/L)
Day 434	P4 + E10; 3 weeks after lorlatinib	340 mg/dL (SI: 8.79 mmol/L)	166 mg/dL (SI: 1.87 mmol/L)	60 mg/dL (SI: 1.55 mmol/L)	245 mg/dL (SI: 6.34 mmol/L)	280 mg/dL (SI: 7.24 mmol/L)
Day 616	P4 + E10 + AH; persistent phase	286 mg/dL (SI: 7.40 mmol/L)	187 mg/dL (SI: 2.11 mmol/L)	53 mg/dL (SI: 1.37 mmol/L)	202 mg/dL (SI: 5.22 mmol/L)	233 mg/dL (SI: 6.03 mmol/L)
Day 987	P4 + E10 + AH; late follow-up before switch	333 mg/dL (SI: 8.61 mmol/L)	182 mg/dL (SI: 2.05 mmol/L)	56 mg/dL (SI: 1.45 mmol/L)	245 mg/dL (SI: 6.34 mmol/L)	277 mg/dL (SI: 7.16 mmol/L)
Day 1043	P4 + E10 + AH; postvisit switch to R10 + E10 + LT4	333 mg/dL (SI: 8.61 mmol/L)	151 mg/dL (SI: 1.70 mmol/L)	55 mg/dL (SI: 1.42 mmol/L)	237 mg/dL (SI: 6.13 mmol/L)	278 mg/dL (SI: 7.19 mmol/L)
Day 1065	R10 + E10 + LT4 + AH; postvisit increase to R15	300 mg/dL (SI: 7.76 mmol/L)	174 mg/dL (SI: 1.96 mmol/L)	54 mg/dL (SI: 1.40 mmol/L)	211 mg/dL (SI: 5.46 mmol/L)	246 mg/dL (SI: 6.36 mmol/L)

Treatment changes listed as postvisit were made after blood sampling at the corresponding visit. Reference ranges are institutional laboratory reference intervals for reporting and differ from guideline-based lipid treatment thresholds for cardiovascular risk management: TC 148 to 248 mg/dL (SI: 3.83-6.41 mmol/L), TG 30 to 117 mg/dL (SI: 0.34-1.32 mmol/L), HDL-C 48 to 103 mg/dL (SI: 1.24-2.66 mmol/L), and LDL-C 65 to 139 mg/dL (SI: 1.68-3.59 mmol/L). Non-HDL-C was calculated as TC minus HDL-C. Because a labeled institutional reference range for non-HDL-C was not available, a calculated reference interval of 45 to 200 mg/dL (SI: 1.16-5.17 mmol/L) was derived from the listed institutional TC and HDL-C reference limits.

Abbreviations: AH, antihypertensive therapy; E10, ezetimibe 10 mg daily; HDL-C, high-density lipoprotein cholesterol; LDL-C, low-density lipoprotein cholesterol; LT4, levothyroxine 25 µg daily; P4, pitavastatin 4 mg daily; R10, rosuvastatin 10 mg daily; R15, rosuvastatin 15 mg daily; TC, total cholesterol; TG, triglyceride.

## Diagnostic assessment

Within 3 weeks after lorlatinib initiation, total cholesterol increased to 340 mg/dL (SI: 8.79 mmol/L), LDL-C to 245 mg/dL (SI: 6.34 mmol/L), triglycerides to 166 mg/dL (SI: 1.87 mmol/L), and HDL-C was 60 mg/dL (SI: 1.55 mmol/L) (Fig. 2A; Table 1). Persistent lipid abnormalities were observed during follow-up. On day 1043, before switching from pitavastatin to rosuvastatin, total cholesterol was 333 mg/dL (SI: 8.61 mmol/L), LDL-C was 237 mg/dL (SI: 6.13 mmol/L), triglycerides were 151 mg/dL (SI: 1.70 mmol/L), HDL-C was 55 mg/dL (SI: 1.42 mmol/L), and non-HDL-C was 278 mg/dL (SI: 7.19 mmol/L).

**Figure 2:**
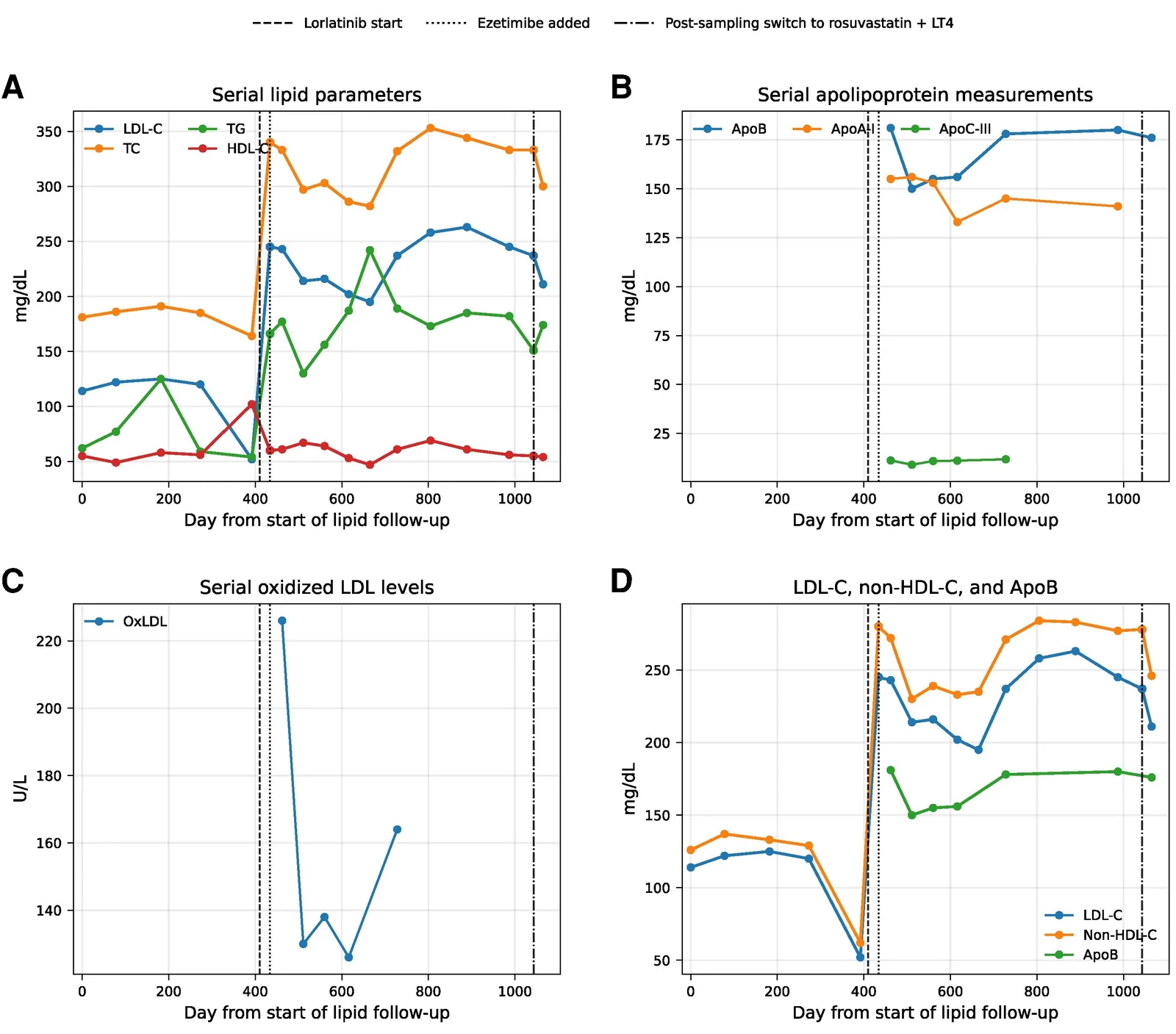
Time course of lipid parameters and apolipoproteins during lorlatinib therapy. (A) Serial changes in total cholesterol, triglycerides, LDL-C, and HDL-C. Day 0 represents the start of lipid follow-up at our outpatient clinic. Vertical lines indicate initiation of lorlatinib on day 410, initiation of ezetimibe on day 434, and the day 1043 visit at which pitavastatin was switched to rosuvastatin and levothyroxine was initiated after blood sampling. (B) Longitudinal changes in ApoB, ApoC-III, and ApoA-I. (C) Serial changes in oxidized LDL. (D) Longitudinal comparison of LDL-C, ApoB, and non-HDL-C.

Representative advanced lipid testing showed ApoB 181 mg/dL (SI: 1.81 g/L) on day 462 and 180 mg/dL (SI: 1.80 g/L) on day 987 (reference range, 66-101 mg/dL [SI: 0.66-1.01 g/L]). Oxidized LDL was 226 U/L on day 462 and 126 U/L on day 616 (reference range, 46-82 U/L). Apolipoprotein C-III (ApoC-III) ranged from 9.0 to 11.8 mg/dL (reference range, 5.4-9.0 mg/dL) and did not progressively increase (Fig. 2B–2D; Table 2). Lipoprotein electrophoretic fractions were available at 5 time points after lorlatinib initiation. The pre-β-fraction remained persistently prominent, ranging from 15.2% to 18.5% (reference range, 6.6-20.8%), while the β-fraction transiently exceeded the upper reference range on day 616 (53.4%; reference range, 33.6-52.0%) (Fig. 3; Table 2).

**Figure 3:**
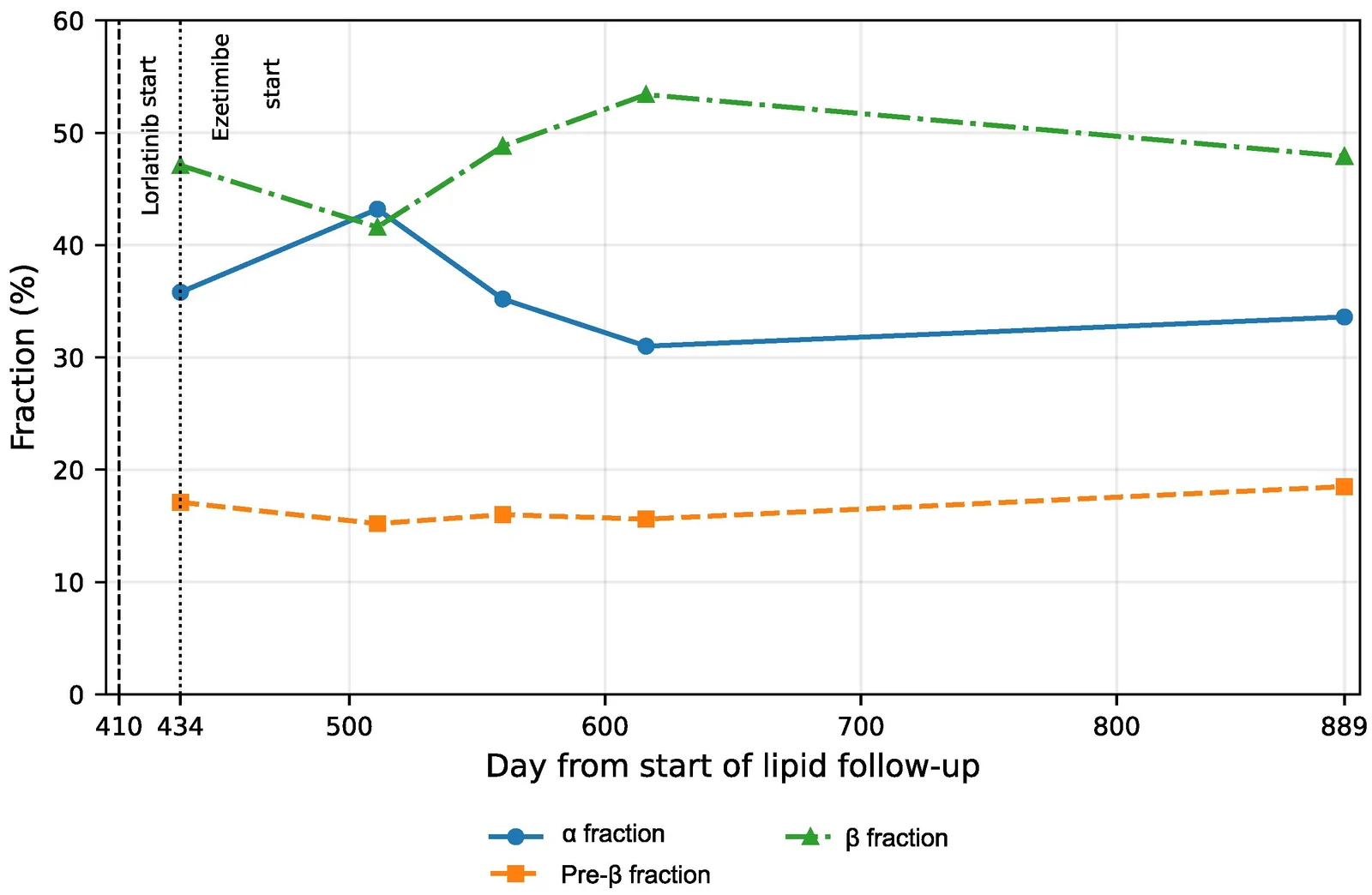
Longitudinal lipoprotein electrophoretic fractions during lorlatinib therapy. Percentages of the α-, pre-β-, and β-lipoprotein fractions measured on days 434, 511, 560, 616, and 889 are plotted. Vertical lines indicate initiation of lorlatinib on day 410 and initiation of ezetimibe on day 434. Reference ranges were 32.6% to 52.5% for the α-fraction, 6.6% to 20.8% for the pre-β-fraction, and 33.6% to 52.0% for the β-fraction.

**Table 2:** Representative qualitative lipoprotein measurements during lorlatinib therapy

Day	Treatment status/change	ApoB (SI)	ApoA-I (SI)	ApoC-III	OxLDL	Fraction α/pre-β/β
Day 434	P4 + E10	NA	NA	NA	NA	35.8%/17.1%/47.1%
Day 462	P4 + E10	181 mg/dL (SI: 1.81 g/L)	155 mg/dL (SI: 1.55 g/L)	11.2 mg/dL	226 U/L	NA
Day 616	P4 + E10 + AH	156 mg/dL (SI: 1.56 g/L)	133 mg/dL (SI: 1.33 g/L)	11.1 mg/dL	126 U/L	31.0%/15.6%/53.4%
Day 889	P4 + E10 + AH	NA	NA	NA	NA	33.6%/18.5%/47.9%
Day 987	P4 + E10 + AH	180 mg/dL (SI: 1.80 g/L)	141 mg/dL (SI: 1.41 g/L)	NA	NA	NA
Day 1065	R10 + E10 + LT4 + AH; postvisit increase to R15	176 mg/dL (SI: 1.76 g/L)	NA	NA	NA	NA

Treatment abbreviations are defined in Table 1. Reference ranges: ApoB 66 to 101 mg/dL (SI: 0.66-1.01 g/L), ApoA-I 126 to 165 mg/dL (SI: 1.26-1.65 g/L), ApoC-III 5.4 to 9.0 mg/dL, OxLDL 46 to 82 U/L, α-fraction 32.6% to 52.5%, pre-β-fraction 6.6% to 20.8%, and β-fraction 33.6% to 52.0%.

Abbreviations: ApoA-I, apolipoprotein A-I; ApoB, apolipoprotein B; ApoC-III, apolipoprotein C-III; NA, not available; OxLDL, oxidized low-density lipoprotein.

Representative baseline laboratory findings did not suggest major hepatic, renal, nephrotic, or glycemic secondary causes of dyslipidemia. Aspartate aminotransferase was 17 U/L (reference range, 13-30 U/L), alanine aminotransferase was 19 U/L (reference range, 7-23 U/L), albumin was 4.4 g/dL (SI: 44 g/L) (reference range, 4.1-5.1 g/dL [SI: 41-51 g/L]), creatinine was 0.59 mg/dL (SI: 52 µmol/L) (reference range, 0.46-0.79 mg/dL [SI: 41-70 µmol/L]), and fasting plasma glucose was 90 mg/dL (SI: 5.0 mmol/L) (reference range, 73-109 mg/dL [SI: 4.1-6.1 mmol/L]). Hemoglobin A1c was 6.1% (SI: 43 mmol/mol), within the prediabetes range (5.7-6.4% [SI: 39-46 mmol/mol]). During follow-up, fasting plasma glucose remained 88 to 93 mg/dL (SI: 4.9-5.2 mmol/L), and hemoglobin A1c ranged from 6.0% to 6.3% (SI: 42-45 mmol/mol), indicating no overt diabetes mellitus requiring treatment.

Thyroid testing using stored serum obtained after lorlatinib initiation showed thyroid-stimulating hormone (TSH) 10.147 mIU/L (reference range, 0.61-4.23 mIU/L) with free thyroxine (FT4) 0.74 ng/dL (SI: 9.5 pmol/L) (reference range, 0.70-1.48 ng/dL [SI: 9.0-19.0 pmol/L]) on day 1023, consistent with subclinical hypothyroidism. On day 1043, TSH was 6.869 mIU/L, FT4 was 0.81 ng/dL (SI: 10.4 pmol/L), and free triiodothyronine was 2.46 pg/mL (SI: 3.78 pmol/L) (reference range, 1.68-3.67 pg/mL [SI: 2.58-5.64 pmol/L]).

## Treatment

Pitavastatin 4 mg daily was continued at lorlatinib initiation. Ezetimibe 10 mg daily was added on day 434 after the marked rise in lipid levels. After repeat lipid and thyroid testing on day 1043, pitavastatin was switched to rosuvastatin 10 mg daily, ezetimibe was continued, and levothyroxine 25 µg daily was initiated after blood sampling. On day 1065, rosuvastatin was increased to 15 mg daily after blood sampling.

## Outcome and follow-up

After approximately 3 weeks of rosuvastatin 10 mg-based therapy and levothyroxine 25 µg daily, follow-up testing on day 1065 showed total cholesterol 300 mg/dL (SI: 7.76 mmol/L), LDL-C 211 mg/dL (SI: 5.46 mmol/L), triglycerides 174 mg/dL (SI: 1.96 mmol/L), HDL-C 54 mg/dL (SI: 1.40 mmol/L), and ApoB 176 mg/dL (SI: 1.76 g/L) (Fig. 2A, 2B, and 2D). Thyroid function testing on day 1065 showed TSH 3.577 mIU/L, FT4 0.85 ng/dL (SI: 10.9 pmol/L), and free triiodothyronine 2.92 pg/mL (SI: 4.49 pmol/L). Because levothyroxine and rosuvastatin were initiated during the same period, subsequent lipid changes could not be attributed to either intervention alone. She continued lorlatinib with sustained tumor shrinkage and no overt cardiovascular events during observation.

## Discussion

This case demonstrates persistent qualitative atherogenic lipoprotein changes during lorlatinib therapy, including sustained elevation of LDL-C, non-HDL-C, and ApoB; persistently increased oxidized LDL; and a prominent pre-β-lipoprotein fraction. Lorlatinib-associated dyslipidemia is clinically well recognized, with hypercholesterolemia and hypertriglyceridemia reported at high frequency [3-5]. The precise mechanism remains incompletely defined. Proposed explanations include increased intestinal lipid absorption, hepatic oversecretion of very-low-density lipoprotein particles, and direct hepatic lipid accumulation; however, these mechanisms remain hypothesis-generating [6, 8].

The longitudinal data did not show a clear discordance among LDL-C, ApoB, and non-HDL-C, because all 3 markers remained persistently elevated after lorlatinib initiation. We therefore interpreted ApoB and non-HDL-C as complementary markers of persistent ApoB-containing lipoprotein burden rather than as evidence of cholesterol-depleted ApoB-rich particles. Persistently increased oxidized LDL levels further supported a potentially more atherogenic profile, but oxidized LDL should be interpreted as a risk-related biomarker rather than proof of a causal mechanism [9, 10].

This case also has implications for lipid-lowering management. Pitavastatin was continued because it had been used before lorlatinib initiation and was tolerated. Pitavastatin, pravastatin, and rosuvastatin are often considered during lorlatinib therapy because of their relatively low potential for clinically relevant CYP3A4-mediated interactions [5]. However, pitavastatin 4 mg daily is generally classified as moderate-intensity therapy. In persistent severe lorlatinib-associated LDL-C or ApoB elevation, earlier escalation to high-intensity rosuvastatin and/or addition of nonstatin therapy, including PCSK9-targeting therapy, should be considered with oncology and cardiology or lipid specialists [11].

Apolipoprotein C-III is an important regulator of triglyceride-rich lipoprotein metabolism through inhibition of lipoprotein lipase activity and reduced hepatic clearance of triglyceride-rich lipoproteins and their remnants [12]. In this patient, however, ApoC-III values were within or near the expected range and did not progressively increase. Therefore, the available data did not support a major role for ApoC-III elevation in explaining the observed phenotype.

In lipoprotein electrophoresis, the pre-β-region generally corresponds to very-low-density lipoprotein and other triglyceride-rich lipoproteins, whereas the β-region corresponds mainly to LDL. Although the measured pre-β-fraction remained within the laboratory reference range, it was consistently in the upper portion of that range across 5 measurements, and the β-fraction transiently exceeded the upper reference range. Electrophoretic fractionation does not provide particle number, particle size, or direct identification of remnant particles. Low-density lipoprotein particle number and particle size were not included in the advanced lipid testing available in our laboratory.

Subclinical hypothyroidism was identified during follow-up and may have contributed to persistent cholesterol-rich lipoprotein elevation. We therefore do not interpret the dyslipidemia as being caused solely by lorlatinib. Nevertheless, the temporal pattern—an abrupt increase in LDL-C and triglycerides within 3 weeks after lorlatinib initiation during ongoing pitavastatin therapy—together with the established association between lorlatinib and dyslipidemia supports lorlatinib as the principal trigger. Because levothyroxine and rosuvastatin were initiated during the same period, subsequent lipid changes cannot be attributed to either intervention alone.

From a monitoring perspective, baseline lipid assessment before lorlatinib initiation and early reevaluation within the first 1 to 2 months are important because lipid abnormalities often occur within the first few weeks [4, 5]. For patients with persistent severe dyslipidemia, prolonged anticipated lorlatinib exposure, or a need for further cardiovascular risk stratification, ApoB and non-HDL-C may provide useful complementary information. Advanced qualitative testing, including oxidized LDL or lipoprotein electrophoresis, should not be routine for all patients but may be informative in selected cases with persistent or complex dyslipidemia.

The main limitations of this report are coexistence of subclinical hypothyroidism during follow-up, lack of LDL particle number and particle size measurements, lack of direct mechanistic experiments, and limited follow-up for cardiovascular outcomes. The absence of overt cardiovascular events should not be interpreted as evidence of low long-term risk. Rather, this case should be considered hypothesis-generating and supportive of careful lipid monitoring, evaluation for potential secondary contributors, and timely treatment intensification during prolonged lorlatinib therapy.

## Learning points

- Lorlatinib-associated dyslipidemia may involve persistent qualitative changes in ApoB-containing and triglyceride-rich lipoproteins beyond isolated LDL-C elevation.
- Apolipoprotein B and non-HDL-C may provide complementary information for assessing persistent atherogenic particle burden during lorlatinib therapy.
- Potential secondary contributors, including thyroid dysfunction, should be evaluated when dyslipidemia persists during lorlatinib therapy.
- In persistent severe lorlatinib-associated dyslipidemia, earlier intensification to high-intensity statin therapy and/or additional lipid-lowering therapy should be considered.

## Contributors

K.S. contributed to the conception and design of the case report, acquisition and analysis of clinical and laboratory data, interpretation of lipid and endocrine findings, and preparation of the manuscript. K.K. contributed to oncologic clinical management, interpretation of the lung cancer treatment course, and verification of the lorlatinib treatment timeline and radiologic response. A.H. contributed to the conception and design of the report, interpretation of the endocrine and lipid findings including secondary causes of dyslipidemia, selection of clinically relevant data, and supervision of the clinical interpretation. All authors approved the final version of the manuscript and agreed to be accountable for all aspects of the work.

## Data Availability

Original data generated and analyzed for this case report are included in this article. No sequence data were generated.
